# Exploring Evolutionary Fitness in Biological Systems Using Machine Learning Methods

**DOI:** 10.3390/e23010035

**Published:** 2020-12-29

**Authors:** Oleg Kuzenkov, Andrew Morozov, Galina Kuzenkova

**Affiliations:** 1 Department of Differential Equations, Mathematical and Numerical Analysis, Lobachevsky State University, 603950 Nizhni Novgorod, Russia; kuzenkov_o@mail.ru (O.K.); kuzenkovagv@mail.ru (G.K.); 2 School of Mathematics and Actuarial Science, University of Leicester, Leicester LE1 7RH, UK; 3 Institute of Ecology and Evolution, Russian Academy of Sciences, 119071 Moscow, Russia

**Keywords:** zooplankton, diel vertical migration, evolutionarily stable strategy, evolutionary fitness, ranking order, machine-learned ranking, pattern recognition

## Abstract

Here, we propose a computational approach to explore evolutionary fitness in complex biological systems based on empirical data using artificial neural networks. The essence of our approach is the following. We first introduce a ranking order of inherited elements (behavioral strategies or/and life history traits) in considered self-reproducing systems: we use available empirical information on selective advantages of such elements. Next, we introduce evolutionary fitness, which is formally described as a certain function reflecting the introduced ranking order. Then, we approximate fitness in the space of key parameters using a Taylor expansion. To estimate the coefficients in the Taylor expansion, we utilize artificial neural networks: we construct a surface to separate the domains of superior and interior ranking of pair inherited elements in the space of parameters. Finally, we use the obtained approximation of the fitness surface to find the evolutionarily stable (optimal) strategy which maximizes fitness. As an ecologically important study case, we apply our approach to explore the evolutionarily stable diel vertical migration of zooplankton in marine and freshwater ecosystems. Using machine learning we reconstruct the fitness function of herbivorous zooplankton from empirical data and predict the daily trajectory of a dominant species in the northeastern Black Sea.

## 1. Introduction

In modern theoretical biology, the concept of long-term selection (natural or human-related) plays a key role in understating outcomes of biological evolution which comes back to the famous idea of “the survival of the fittest” by C. Darwin [1]. The fundamental idea consists in introducing a quantity called evolutionary fitness which can describe selective advantages of inherited elements in the underlying biological system (e.g., genotype, life history trait, behavior, etc.). Maximization of fitness would provide the eventual outcomes of long-term biological evolution [2,3,4,5]. The concept of evolutionary fitness was first developed in the works of Haldane, Fisher, and Wright [6,7]. In particular, Wright visualized biological evolution as a “hill-climbing” process that increases the fitness of a given population in a landscape of inherited elements [2,7,8]. However, at the same time, evolutionary fitness has always been a controversial issue as different authors have proposed distinct definitions of fitness [9,10,11,12]. There is a discussion in the literature on how one can rigorously define evolutionary fitness [3,13,14]. For example, it was argued that fitness can be defined as some generalized entropy [15]. However, the problem is still far from its final solution. Note that different definitions of fitness may produce conflicting predictions of evolutionary outcomes [14,16].

Recently, a new mathematically rigorous framework has been developed to quantify fitness in biological systems [17,18,19]. The idea was inspired by an early paper by A. Gorban [20]. The framework considers the outcomes of long-term dynamics of a large number of competing inherited elements which we will call strategies (they can be behavioral strategies, life history traits, genotypes, etc.). The strategies are ranked according to their competitive advantages [21]. Fitness is mathematically defined as a certain function which preserves the introduced ranking of strategies. The major challenge of implementing the above concept of fitness is to how to mathematically express the fitness function via parameters describing life history traits (or behavioral patterns) of organisms. Deriving an analytical expression for evolutionary fitness is possible for some classes of models of population dynamics, including those accounting for age structure [22]. The approach developed by A. Gorban [20] was applied to several problems ranging from preventing the tragedy of the commons to cancer therapy [23].

However, the parameters and coefficients in a mathematical model of population dynamics are hard to estimate empirically, which makes predicting the evolutionary outcomes from the model complicated or even impossible. A possible solution to avoid this obstacle is to construct evolutionary fitness using the observed population dynamics, for example, the time series containing empirical information on ranking of strategies (e.g., which strategy is more advantageous). In this case, the problem of reconstruction of fitness in biological systems will be a special case of the generic problem of ranking elements. Indeed, mathematically the general problem of ranking elements of some set is defined as follows. A partial order is defined on a certain finite subset of elements of some compact metric subspace (this subset can be considered as the training data set): one needs to extend it over the entire space without changing the ranking of the initial (training) subset of elements. In this paper, we address the problem of ranking of some inherited elements by using a pairwise comparison approach. Ranking can be often defined with the help of a comparison function, *J*, in the considered space, where better (fitter) inherited elements would correspond to higher values of *J*. In this case, the function *J* can be understood as fitness.

Among other approaches, machine learning methods seem to be very promising for establishing ranking of elements of different nature, in particular, related to inherited strategies or life history traits in biological systems [24,25,26]. Therefore, it would be natural to implement the existing techniques of machine learning to reconstruct the fitness function from empirical data or a mathematical model. In [27,28], it was shown that ranking of strategies and identifying the fitness function is similar to the problem of classification and separation of ordered pairs of elements into two main categories: “the first element is better than the second one” and “the second element is better than the first one”. Note that artificial intelligence systems are currently being implemented in pattern recognition in computational biology [29,30]. In the current paper, we demonstrate that this methodology can be implemented to explore the optimal patterns of diel vertical migration (DVM) of zooplankton.

The phenomenon of diel vertical migration (DVM) of aquatic organisms such as zooplankton was discovered long ago [10,11,12,31]. In particular, it was shown that a large number of zooplankton species move up and down between deep and surface layers on a daily basis [32]. DVM of zooplankton plays a crucial role in the dynamics of the organic matter of the ocean, and this phenomenon is regarded as the greatest synchronous migration of biomass on Earth [31,32]. Identifying the causes and mechanisms of DVM of zooplankton is an important problem in modern ecology and is crucial for an efficient fishery. The underlying mechanisms of DVM, as well as its ecological significance, have been largely studied both empirically and theoretically [4,10,11,12,16]; however, many aspects of this phenomenon are still poorly understood. In particular, this is due to the wide variety of patterns of DVM observed in nature. Several mathematical models of DVM were suggested in the literature, most of them are focused on the optimization of some particular criterion which was somewhat arbitrary chosen depending on a study [9,10,11,12,16,33]. In this paper, we use the data from the empirical observations of zooplankton DVM in the northeastern Black Sea to demonstrate how evolutionary fitness and optimal DVM can be revealed using artificial intelligence systems and machine learning. We emphasize that similar computational approaches can be implemented to explore fitness in other (non-planktonic) biological systems as well.

## 2. Materials and Methods

### 2.1. Generic Framework to Estimate Evolutionary Fitness

Here, we briefly introduce a mathematical framework to rigorously quantify evolutionary fitness in self-replicating systems with strong inheritance. Note that more details on defining evolutionary fitness in mathematical models are provided in [5,19,22]. We formally introduce the space of hereditary strategy *V* of a certain population. The space *V* includes all possible strategies *v* which can be considered as behavioral strategies or/and life history traits. We assume *V* to be metric. Let each strategy *v* at each moment of time *t* be associated with some non-negative value ρ(v,t), which quantifies its presence in the self-replicating system. In the simplest case, ρ(v,t) is the number of individuals realizing strategy *v*; this can be also understood as the density distribution of the population over *V*. We assume that ρ(v,t) the following properties hold:

1. ρ(v,t)=0 signifies the absence of *v* in the system at time t;
2. ρ(v,t)>0 signifies the presence of *v* in the system at time t;
3. ρ(v,t) is a continuous function of *v* in V;
4. ρ(v,t) is a continuous function of time;
5. approaching zero by ρ(v,t) over time means the extinction of the strategy v;
6. if $\rho (v,t_{0})=0$ at some time $t_{0}$, then ρ(v,t)=0 for all $t>t_{0};$ and
7. ρ(v,t) is uniformly bounded by a constant, i.e., ρ(v,t)<C.

The above-introduced function ρ(v,t) is a general mathematical description of the self-replicating system, for example, it can be defined via a certain mathematical model (e.g., based on a system of differential equations). Thus, a self-replicating system is a pair (V,ρ(v,t)), satisfying above the assumptions.

We can formally define ranking of strategies as follows. The strategy *v* is better than strategy *w* (symbolically we denote this as v≻w) in the ratio ρ(w,t)/ρ(v,t) tends to zero with time, i.e.,

(1) $$\lim_{t\to \infty }\frac{\rho (w,t)}{\rho (v,t)}=0.$$

The above definition means that the subpopulation with strategy *v* goes extinct with t→+∞. Thus, the ranking order is introduced in *V*.

We assume there exists a functional J(v) that preserves the ranking of the strategies, i.e.,

(2) J(v)>J(w)⇔v≻w.

The functional *J* can be referred to as evolutionary fitness. In general, the ranking order, and thus *J*, may depend on the initial condition (e.g., systems with cyclic ranking order, see in [17,19,34]). Note that dealing with scenarios, where *J* depends on initial condition, is, in general, a challenging mathematical task [5]. For this reason, in this study, we make the simplifying (but restrictive) assumption that evolutionary fitness does not depend on the initial condition. In this case, *J* plays the role of a ranking function of in the system. Mathematically, the existence of the above-introduced fitness is subject to the requirements imposed by the Debreu theorem on continuity (i.e., in the case v≻w, we require the same ranking to hold between all strategies which are sufficiently close to *v* and *w*) and rationality of *v* [35]; in this case *J* is a continuous function. From the biological point of view, the requirements of rationality and continuity of strategies (or life history traits) *v* seem to be natural, so we assume that they hold. The strategy $v^{*}$ for which evolutionary fitness attains its maximum will be the optimal one in the sense that this strategy will eventually outcompete all others for which fitness is lower.

For each v, the temporal dynamics of ρ(v,t) is usually determined by a few parameters (their total number is *n*) such as foraging rates, maturation times, mortality rates, reproduction, etc., each of which is a function of the strategy *v*. The set of those parameters can be described by a vector $M\left(v\right)=(M_{1}\left(v\right),M_{2}\left(v\right),\ldots ,M_{n}\left(v\right))$. Evolutionary fitness *J* will be a multivariable function of M, i.e., J=J(M(v)).

We assume that we can approximate *J* using Taylor expansion around a certain point $M_{0}$. In particular, we can consider the following algebraic form of degree *k* (i.e., *k* is the maximal power in the Taylor expansion)

(3) $$J\approx F\equiv \sum_{1\leq i_{1}+i_{2}+\ldots +i_{n}\leq k}\lambda_{i_{1}i_{2}\ldots i_{n}}M_{1}^{i_{1}}M_{2}^{i_{2}}\ldots M_{n}^{i_{n}},$$

where $i_{j}$ are non-negative integer numbers and $0\leq i_{j}\leq k$, 1≤j≤n, and $\lambda_{i_{1}i_{2}\ldots i_{n}}$ are the coefficients of the Taylor expansion.

In our notation, *F* will denote an approximation of fitness J. The approximation *F* is simpler to explore than the original function *J*; however, it still might be complicated to estimate the coefficients in the above Taylor expansion. Below, we propose a computational method of estimating the coefficients in (3) using modern methods of pattern recognition. The crux of our computational method to reconstruct fitness is the following [27,28].

We introduce the following notation for the products of $M_{i}$

$$Q_{i_{1}i_{2}\ldots i_{n}}=M_{1}^{i_{1}}M_{2}^{i_{2}}\ldots M_{n}^{i_{n}},\mathrm{where}1\leq i_{1}+i_{2}+\ldots +i_{n}\leq k.$$

Let *P* be an ordered set of $Q_{i_{1}i_{2}\ldots i_{n}}$, i.e.,

$$P=\{Q_{i_{1}i_{2}\ldots i_{n}}1\leq i_{1}+i_{2}+\ldots +i_{n}\leq k,0\leq i_{j}\leq k,1\leq j\leq n\}.$$

The entire set of *P* forms a vector space. The approximation of fitness provided by (3) becomes a linear function of $Q_{i_{1}i_{2}\ldots i_{n}}$, i.e., the coordinates of the vector space *P*.

We next consider a pair of strategies (v,w) and the corresponding approximations of the fitness functions related to those strategies. In the case, where *v* is better than w, we have F(M(v))>F(M(w)), i.e.,

$$\sum_{1\leq i_{1}+i_{2}+\ldots +i_{n}\leq k}\lambda_{i_{1}i_{2}\ldots i_{k}}\left[Q_{i_{1}i_{2}\ldots i_{n}}\left(v\right)-Q_{i_{1}i_{2}\ldots i_{n}}\left(w\right)\right]>0.$$

In the case where *w* is better than v, we have F(M(w))>F(M(v)).

We next assign the pair of strategies (v,w) to the difference (P(v)−P(w)) and the pair (w,v) to the difference (P(w)−P(v)). In the vector space *P* we consider the hyperplane given by

(4) $$Y_{k}\equiv \sum_{1\leq i_{1}+i_{2}+\ldots +i_{n}\leq k}\lambda_{i_{1}i_{2}\ldots i_{n}}Q_{i_{1}i_{2}\ldots i_{n}}=0.$$

Hyperplane (4) separates the sets of points in the vector space *P* with coordinates $Q_{i_{1}i_{2}\ldots i_{n}}$ with the two different ranking order generated by underlying strategies. The normal vector of this hyperplane provides the coefficients $\lambda_{i_{1}i_{2}\ldots i_{n}}$ in the approximation of fitness F, i.e., $Y_{k}$ will be essentially a Taylor approximation of order *k* of evolutionary fitness.

The problem of separation of two or more sets of points with distinct properties is a typical pattern recognition problem (known as linear classification) that can be solved with the help of learning neural networks [36,37,38]. The problem essentially boils down to determining whether the ordered pairs of elements “first, second” belong to one of two classes: “the first is better than the second” or “the first is worse than the second”. To separate two sets (domains) of pairs with different ranking order, we can use either the classifier based on Fisher’s determinant, the nearest neighbor method or an artificial neural network [38]. In particular, using several test examples, we conducted a brief comparison of the efficiency of the nearest neighbor method and a single-layer artificial neural network. We observed that the accuracy of recognition for the neural network is higher than that of the nearest neighbor method (98% against 95%). Moreover, using artificial networks technology is more advantageous for the given problem since the weight coefficients of individual neurons of the first layer of the network allow us to determine the coefficients of the separating hyperplane $Y_{k}=0$, i.e., they give the coefficients in the Taylor approximation of fitness. Note that another potential approach to solve the problem of identification of parameters of fitness is linear programming. Linear programming was previously applied to the problem of fitness identification [5]. However, linear programming methods have several disadvantages: they are very sensitive to errors in data even if such errors are small. The presence of even small errors in data often makes the system of linear inequalities inconsistent, and the chance of having inconsistency increases with the overall number of data points. On the other hand, a neural network can efficiently work even in the presence of small errors in data. Finally, an increase in the amount of data usually requires rewriting software using linear programming, whereas it is not the case of neural networks.

Note that separation of two finite sets of points from the learning sample with the hyperplane $Y_{k}=0$ is generally non-unique. The concrete realization of the separation procedure (and the corresponding approximation of fitness) depends on the method of constructing the separating hyperplane. The location of this hyperplane can be slightly different depending on the methods used in neural networks. In the case where one implements a simple single-layer neural network, constructing a separating hyperplane will be equivalent to the gradient descent for a certain function depending on the coefficients of the hyperplane. There also exists the concept of an optimal hyperplane which can be constructed using, for example, the Gauss–Seidel method. The problem of fitness reconstruction can also be solved using pointwise methods of machine learning ranking, for example, OPRF (polynomial regression) [39].

Finally, after estimating the coefficients in the approximation of evolutionary fitness, one can derive the evolutionarily stable (optimal) strategy by the methods of calculus of variations or the optimal control theory. Here, the term “evolutionarily stable” signifies that such a strategy cannot be invaded by a different strategy in the course of natural selection: all the other strategies would be eventually eliminated [4]. In this case, the fitness function will be the objective functional of the corresponding optimizing problem [21,22]. We have designed particular software able to solve this problem numerically for any known objective functional [40,41].

In this paper, we verify the efficiency of the proposed framework or some models where the fitness function is can be derived analytically. As an important study case, we explore optimal trajectories of diel vertical migration of herbivorous zooplankton in the ocean and lakes, the phenomenon, which is considered to be the most significant synchronous biomass movement on Earth.

### 2.2. Predicting Patterns of Optimal DVM via Machine Learning

It was previously shown, using both empirical data and mathematical modeling, that the timing and amplitude of DVM of herbivorous zooplankton are mainly determined by the following environmental factors [22,42]: the density of food E(x) (phytoplankton) at the depth *x* of the column, the distribution of visual predators $S_{x}\left(x\right)$ consuming zooplankton (e.g., fish), and their daily activity $S_{t}\left(t\right),$ other factors resulting to zooplankton mortality such as the unfavorable temperature or/and hydrogen sulfide concentration [10,16]. All of these factors can be considered as mathematical functions of the vertical coordinate *x* and the time of day *t* (the time of day is rescaled, so t=1 corresponds to 24 h). We suggest that environmental characteristics do not evolve.

We can formally consider the daily trajectory x(t) of a zooplankton grazer in the column to be its strategy. Note that herbivorous zooplankton have various developmental stages characterized by different life traits and distinct patterns of DVM. In this case, the inherited strategy *v* of a subpopulation is defined mathematically as a set of *N* periodic functions $X\left(t\right)=(x_{1}\left(t\right),\ldots x_{N}\left(t\right)),$ where *N* is the number of considered in the model developmental stages of zooplankton. Note that for simplicity we can combine several biological stages in a single one to simplify the model. We assume that every $x_{i}\left(t\right)$ is a continuously differentiable function on the segment [0,1], satisfying conditions $x_{i}\left(0\right)=x_{i}\left(1\right)$.

One substantial obstacle for identification of parameters of the fitness function using neural networks techniques is the lack of a sufficient amount of empirical data necessary to create a learning sample. Often ecological observation is focused on systems with only a small number (e.g., dozens) of competing species described by distinct life history traits or strategies. This number is often too small to guarantee efficient learning of a proper neural network. Here, we suggest the following solution to the problem. We assume that the observed strategy or life history trait is the result of long-term selection, thus can be considered to be evolutionarily stable under the given environmental conditions. Thus, deviation from this strategy would reduce evolutionary fitness. Mathematically, the above assumption can be formulated as follows.

Consider that the observed strategy (trajectory) of diel vertical migration is evolutionarily stable, so it realizes the maximum of fitness *F*. This strategy is described by the trajectory $x^{*}\left(t\right)$. We may consider an arbitrary function y(t) satisfying y(0)=y(1) and some positive ε, so the deviation from the optimal trajectory will be εy(t). In this case, we have for fitness $J\left(x^{*}\left(t\right)\right)>J\left(x^{*}\left(t\right)+\varepsilon y\left(t\right)\right)$. For sufficiently small ε>0 (i.e., in the neighborhood of $x^{*}\left(t\right))$, using a smaller in absolute value ε will result in a higher value of fitness, i.e., for $|\varepsilon_{1}|>|\varepsilon_{2}|>0$ we have $J\left(x^{*}\left(t\right)+\varepsilon_{1}y\left(t\right)\right)>J\left(x^{*}\left(t\right)+\varepsilon_{2}y\left(t\right)\right)$. This assumption allows us to generate a large number of samples necessary to guarantee a successful machine learning process.

The above approach can be realized as follows. The observed strategy is set as a vector of vertical positions of an organism at some moments throughout the day. Next, we modify some components of this vector by adding to them some constants multiplied by ε. As a result, we obtain new vectors of daily vertical locations of zooplankton. We may assume that an artificially generated strategy is negatively correlated with the absolute value of ε. The generated samples are added to improve the learning sample.

To explore optimal patterns of DVM, we used the empirical data collected in the northeastern Black Sea. The details about the data are provided in [42]. We considered data on DVM in summer 2011 for migration of a dominant herbivorous copepod *Calanus euxinus*. To describe the vertical distribution of predators and their activity throughout the day, we used the data from in [10,16].

Technically, we used the following programming software: machine learning library Scikit-learn for Python; library Pandas (This is a programming library based on Python for processing and analysis of data); module CSV (comma-separated value), which allows one to work with the same format of tables (it is needed for correct reading of the input data from a file with the extension .csv); and library Numpy, which allows one to work with large multi-dimensional arrays and matrices, as well to implement functions to quickly process multidimensional arrays and matrices.

## 3. Results

### 3.1. Revealing Fitness in a Non-Structured Population

We firstly consider a non-structured population of zooplankton, i.e., we neglect the difference of individuals in terms of their developmental stages, and disregard the maturation effects. As explained in Section 2, fitness function can be constructed using the coefficients of the separating hyperplane $Y_{1}\left(P\right)=0$ defined by (4). Note that previous studies using empirical data of DVM of herbivorous zooplankton indicate that the following four (macro)-parameters have the greatest impact on population success of grazers [19,42]:

$$M_{1}\left(v\right)=\int_{0}^{1}E\left(x\left(t\right)\right)dt,\;\;M_{2}\left(v\right)=-\int_{0}^{1}S_{t}\left(t\right)S_{x}\left(x\left(t\right)\right)dt,$$

$$M_{3}\left(v\right)=-\int_{0}^{1}{\left[x^{\prime }\left(t\right)\right]}^{2}dt,\;\;M_{4}\left(v\right)=-\int_{0}^{1}G\left(x\left(t\right)\right)dt.$$

The above parameters have the following transparent biological meaning. $M_{1}$ is the amount of consumed food (phytoplankton) per day; $M_{2}$ is the daily mortality due to visual predation (e.g., by fish); $M_{3}$ is the metabolic cost of vertical migration (we assume it to be proportional to the losses due to resistance forces or drag); $M_{4}$ is an increase in mortality of zooplankton when entering surface or deep water which are characterized by unfavorable temperature and hydrogen sulfide conditions [42]. In the above expressions, t=1 corresponds to 24 h.

Following previous studies [19], we approximate evolutionary fitness of a non-structured population with the simplest linear function(al), which is a weighted sum of the terms $M_{i}$,

(5) $$F\left(v\right)=\alpha M_{1}\left(v\right)+\gamma M_{2}\left(v\right)+\beta M_{3}\left(v\right)+\delta M_{4}\left(v\right),$$

where α,β,γ,δ are unknown positive coefficients which have the same meaning as $\lambda_{i}$ in (3). These coefficients quantify the degree of impact of each particular factor $M_{i}$ on fitness. To fully specify fitness in the above model, one needs to evaluate α,β,γ,δ from available data (note that only three of them are independent).

It is important to stress that the linear form of fitness (5) can arise in some nonlinear models of population dynamics, for example, they can include effects of intraspecific competition [19], and an example of such models is given in Appendix B. On the other hand, to derive the optimal trajectory of DVM of zooplankton one can solely use expression (5) for fitness combined with available data on the ranking of DVM trajectories, i.e., just keeping in mind that some underlying mathematical model exists. As such, in this subsection, we will reveal the optimal DVM trajectory exclusively using (5) and data.

In practice we have only discrete values of the rates E,S, and *G* measured at some particular moment of the day as it is impossible to continuously collect zooplankton samples across the water column (in this study the sampling interval was 3 h, see [42]). Therefore, we should formally use a discrete version (approximation) of the above integrals. This gives

$$F=\alpha \sum_{l=1}^{L}E\left(x\left(t_{i}\right)\right)-\beta \sum_{l=1}^{L}S\left(x\left(t_{i}\right),t_{i}\right)-\gamma \sum_{l=1}^{L}{\left(x^{\prime }\left(t_{i}\right)\right)}^{2}-\delta \sum_{l=1}^{L}G\left(x\left(t_{i}\right)\right).$$

In the above expression, we considered L=8 data time points for the zooplankton positions during the day following the observation data from in [42]. Note that time increment $\Delta t=t_{i+1}-t_{i}=3h$ is constant (for simplicity, we do not multiply each term in the above expression by the same multiplier Δt). In Appendix A, we provide details on the AI procedure used for comparison of two different strategies of zooplankton DVM based on empirical data.

We start solving the problem of fitness identification from data with simple functional parameterizations of E,S, and *G* given by a linear and quadratic functions. In other words, we consider $E=\sigma_{1}\left(x+C\right),S_{x}=\sigma_{2}\left(x+C\right),-C<x<0;G={\left(x+C_{0}\right)}^{2},S_{t}=\cos\left(2\pi t\right)+1,0<t<1,$ where *C* and $C_{0}$ are some characteristic depths. This approach seems to be logical as it allows us to obtain a reasonable first approximation of the optimal DVM trajectory and coefficients α,γ,β,δ which can differ by several orders of magnitude: this knowledge will be essential when considering more complicated parameterizations of E,S and G. On the other hand, we can easily verify the efficiency of the method since for the considered E,S and *G* one can derive an analytic expression for the optimal strategy. Using available data (see, e.g., in [42]), we can roughly estimate the parameters $C,\sigma_{1},\sigma_{2},C_{0}$ in the above simple parameterization of E,S and *G*. For example, considering $C=140\;m,C_{0}=60\;m,\sigma_{1}=0.25,\sigma_{2}=0.003$ will be in accordance to empirical data [42]. The above linear and quadratic functions should be regarded as the simplest approximations of the realistic nonlinear parameterizations of E,S, and *G*, which are described in detail further in this subsection.

It was demonstrated in [19] that for the considered linear and quadratic parameterizations of E,S, and G, the optimal solution (realizing the maximum of the functional (5)) will be given by $x^{*}\left(t\right)=A+B\cos\left(2\pi t\right),$ where the constant mean depth through the day *A* is determined by $A=\left(\sigma_{1}\alpha -\sigma_{2}\gamma \right)/2\delta -C_{0}$ and the amplitude of vertical migration *B* is determined by $B=-\sigma_{2}\gamma /\left(2\delta +2{\left(2\pi \right)}^{2}\beta \right)$. The integral parameters $M_{i}$ can be expressed as $M_{1}=\sigma_{1}\left(A+C\right),M_{2}=-\sigma_{2}\left(A+C+B/2\right),M_{3}=-{\left(2\pi \right)}^{2}B^{2}/2,$ and $M_{4}=-\left({\left(A+C_{0}\right)}^{2}+B^{2}/2\right)$. The obtained analytical solution can then be compared with the available empirical data.

We used artificial neural network methods to find the coefficients α,γ,β,δ. For the training set, we used 202 different strategies and 2031 pairs. The considered sample consisted of the learning part (70%) and the part used for testing (30%) with the help of the module train_test_split from the library sklearn.model_selection. To evaluate the efficiency of learning, we used Logloss, which was very low (logloss =0.01701). We also use another way to verify the efficiency of learning based on the function cross_val_score from the library sklearn.model_selection. In this case, the success of object detection is 99.89%.

**Figure 1:**
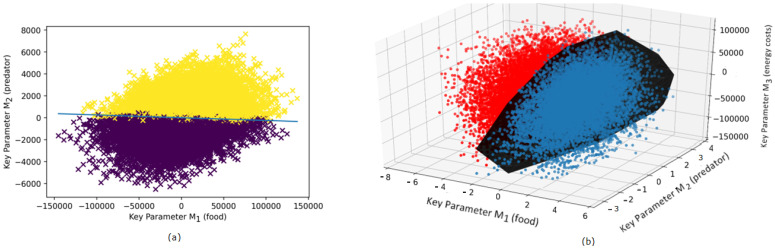
Revealing evolutionary fitness in an unstructured (single-stage) zooplankton DVM model by separating pair strategies with the different ranking order. (**a**) Two-dimensional cross section $(M_{1}\left(v\right)-M_{1}\left(w\right)$, $M_{2}\left(v\right)-M_{2}\left(w\right))$ of the four-dimensional parameter space $\left\{M_{i}\left(v\right)-M_{i}\left(w\right)\right\}$, i=1,2,3,4. (**b**) Three-dimensional cross section $(M_{1}\left(v\right)-M_{1}\left(w\right)$, $M_{2}\left(v\right)-M_{2}\left(w\right)$, $M_{3}\left(v\right)-M_{3}\left(w\right))$ of the four-dimensional parameter space. In both figures, each dot represents the difference $M_{i}\left(v\right)-M_{i}\left(w\right)$ (or equivalently, $M_{i}\left(w\right)-M_{i}\left(v\right))$ and corresponds to the pair strategies (v,w). In both figures, dark dots denote pairs with w≻v and bright dots denote pairs where w≺v. The considered key parameters denote food availability $(M_{1}),$ predator pressure $(M_{2}),$ and the metabolic cost of migration $\left(M_{3}\right)$. The strategies are obtained from empirical data on vertical trajectories of DVM of *Calanus euxinus* (adult stage) in the northeastern Black Sea in the summer period (see Section 2 for detail).

The result of the comparison of pairs of strategies (trajectories of DVM) based on their relative ranking (i.e., w≻v or w≺v) is shown in Figure 1. As the dimensionality of the key parameter space $M_{i}$ is n=4, we only show two examples of cross sections in 2D and 3D spatial settings by fixing the remaining parameters. Each point in the diagram represents a pair of strategies (more precisely, we plot the differences between the parameters calculated as $(M_{i}\left(v\right)-M_{i}\left(w\right)$). In Figure 1a, we show the strategies in the plane $\left(M_{1}\left(v\right)-M_{1}\left(w\right),M_{2}\left(v\right)-M_{2}\left(w\right)\right)$. Two different colors of the plotted points correspond to the two possible outcomes of ranking, i.e., either w≻v or w≺v. The straight line separates the points with different rankings. Figure 1b shows a 3D cross section of the parameters $\left(M_{1}\left(v\right)-M_{1}\left(w\right),M_{2}\left(v\right)-M_{2}\left(w\right),M_{3}\left(v\right)-M_{3}\left(w\right)\right)$ by fixing $M_{4}$. The black hyperplane separates the pair points with different ranking order. Note that variation of the fourth parameter $M_{4}$ shows in a similar pattern as those shown in Figure 1, so we do not present here the corresponding diagrams for brevity. We found that the above cross section patterns are not very sensitive to the choice of the remaining parameters unless these parameters are taken from peripheral parts of the region of data points.

**Figure 2:**
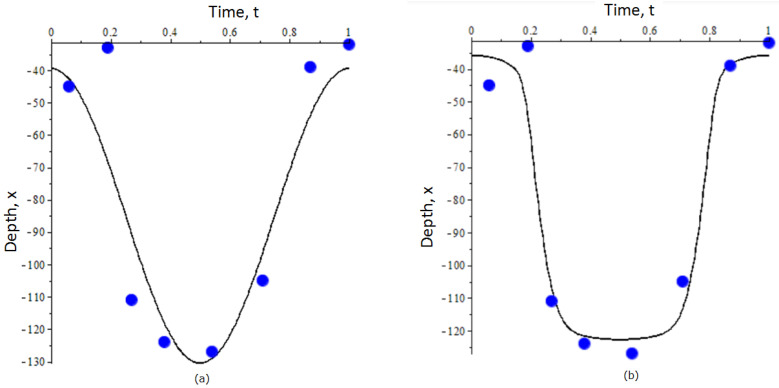
Optimal trajectories of DVM of zooplankton predicted by an unstructured (single-stage) population model: (**a**) $E,S_{x},$ and *G* in $M_{i}$ are modeled by the simple linear and quadratic parameterization; (**b**) $E,S_{x},$ and *G* are modeled by hyperbolic functions. Blue dots show the empirical data of vertical migration of *Calanus euxinus* in the northeastern Black Sea in summer (adult stage) [42]. For details on the construction of trajectories and parameterization of $E,S_{x},$ and *G* see the text. The depth *x* in the graphs is measured in meters.

By separating the pairs of strategies with distinct ranking order (see Figure 1), we obtained the following estimates for the parameters α,γ,δ, and β: α=1.96, $\gamma =3.3\cdot 10^{2}$, $\delta =10^{-2}$, and $\beta =2.5\cdot 10^{-5}$ in the fitness function. Then, using the provided above analytical expressions for *A* and *B*, we derive the mean depth and the amplitude of migration: A=80.16 m and B=45.0 m. In Figure 2a, we plot the DVM trajectory (dashed line) along with the empirical data which we used in this study [42].

We further explored DVM of zooplankton under a more realistic parameterization of $E,S_{x}$, and G, in particular the one based on hyperbolic functions [19]. We consider that $E\left(x\right)=\sigma_{1}(\tanh\left(\xi_{1}\left(x+C1\right)+1\right),S_{x}\left(x\right)=\sigma_{2}\left(\tanh\xi_{2}\left(x+C1\right)+1\right),$ and $G\left(x\right)=\cosh(\xi_{3}\left(x+C_{0}\right))$ with model parameters $C=140\;m,C_{0}=80\;m,C_{1}=40\;m,\sigma_{1}=0.25,\sigma_{2}=0.003,\xi_{1}=\xi_{2}=0.025\frac{1}{m},$ and $\xi_{3}=0.2\frac{1}{m}$. For the daily activity of visual predators $S_{t}$ we use the same parameterization as before, i.e., $S_{t}=\cos\left(2\pi t\right)+1$. Note that the parameterization of the vertical distribution of food E(x) in the form of tanh is justified by empirical observation [42], the same concerns the vertical distribution of visual predation load S(x) [16]. The parameterization of the natural mortality G(x) is in fact an approximation of the sum of two sigmoidal functions used in [42] which has an empirical justification. The values of parameters functions in E,S,G are taken close to those considered in [42].

Implementation of artificial neural networks for the above-mentioned parametrization gives the following values for the coefficients in (5): $\alpha =2.5,\gamma =3.33\cdot 10^{2},\beta =7.5\cdot 10^{-9}$, and $\delta =10^{-5}$. The figures separating ranking order of pairs of strategies are similar to those shown in Figure 1 and thus are not presented here. Using the obtained values of α,β,γ,δ, we derived the optimal trajectory x(t) of zooplankton DVM, which is shown in Figure 2b. Mathematically, we apply the standard methodology of the optimal control based on the Pontryagin’s maximum principle (see in [19] for details).

### 3.2. Revealing Evolutionary Fitness in a Structured Population

We can improve our prediction of the optimal DVM by considering a more realistic situation with age structuring in the zooplankton population. It is known that different developmental stages may show distinct patterns of migration, in particular, the earliest stages of marine copepods usually do not show a pronounced migration [12,16,42]. Note that herbivorous mesozooplankton such as the *Calanus* genus are often characterized by six distinct developmental stages; however, for the sake of simplicity, here we combine some stages and consider only two categories: juveniles and adults. The modeled strategy of DVM is now described by a vector $(x_{1}\left(t\right),x_{2}\left(t\right)),$ which describes the daily change of the depth by (trajectory) of a juvenile and an adult grazer, respectively. As previously, we assume that fitness of each developmental stage is determined by only four main factors (food consumption, predation losses, the metabolic cost of migration, and extra mortality at unfavorable depths), so the resultant number of parameters now increases up to eight:

$$M_{1}\left(v\right)=\int_{0}^{1}E\left(x_{1}\left(t\right)\right)dt,\;\;M_{2}\left(v\right)=-\int_{0}^{1}S_{t}\left(t\right)S_{x}\left(x_{1}\left(t\right)\right)dt,$$

$$M_{3}\left(v\right)=-\int_{0}^{1}{\left[x_{1}^{\prime }\left(t\right)\right]}^{2}dt,\;\;M_{4}\left(v\right)=-\int_{0}^{1}G\left(x_{1}\left(t\right)\right)dt,$$

$$M_{5}\left(v\right)=\int_{0}^{1}E\left(x_{2}\left(t\right)\right)dt,\;\;M_{6}\left(v\right)=-\int_{0}^{1}S_{t}\left(t\right)S_{x}\left(x_{2}\left(t\right)\right)dt,$$

$$M_{7}\left(v\right)=-\int_{0}^{1}{\left[x_{2}^{\prime }\left(t\right)\right]}^{2}dt,\;\;M_{8}\left(v\right)=-\int_{0}^{1}G\left(x_{2}\left(t\right)\right)dt.$$

which have the same meaning as previously. Note that the parameters $M_{1},M_{2},M_{3},M_{4}$ are related to juveniles, whereas $M_{5},M_{6},M_{7},M_{8}$ characterize the adult stage.

We construct the fitness function based on the following quadratic form (the quadratic terms are needed to make the interconnection between the two stages, for example, via maturation of juveniles and reproduction of adults),

(6) $$F=\sum_{i=1}^{8}\lambda_{i}M_{i}+\sum_{i=1}^{8}\sum_{j=1}^{8}\lambda_{ij}M_{i}M_{j},$$

where the parameters $M_{i}$ are (as before) time discrete approximations (measured every 3 h) of the corresponding integrals (here $\lambda_{ij}=\lambda_{ji}$). The choice of *F* in the form (6) for a two-stage population is confirmed by analytical results based on a system of differential equations (see Appendix B for detail).

**Figure 3:**
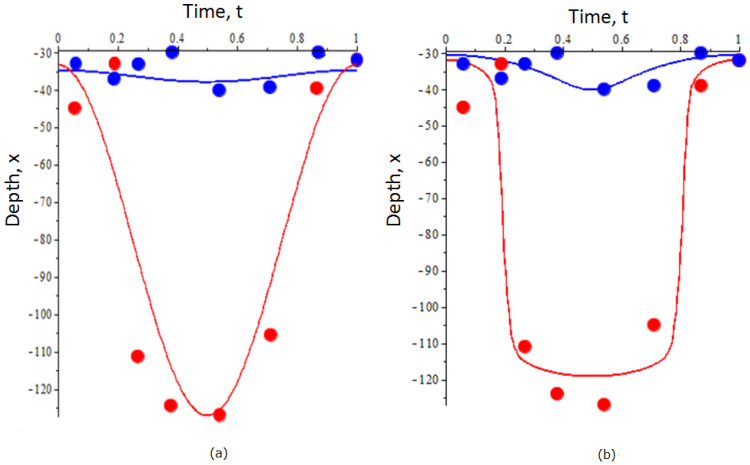
Optimal trajectories of DVM of zooplankton predicted by a two-stage model in the case where the functions *E*, $S_{x}$, and *G* are the given by linear and quadratic functions (**a**) and in the case where *E*, $S_{x}$, and *G* are given by hyperbolic functions (**b**). In both figures, the dots show the empirical data of vertical migration of *Calanus euxinus* in the northeastern Black Sea in summer from [42]: blue and red color denote, respectively, juveniles and adults. The blue and red lines denote the optimal trajectories for juveniles and adults, respectively, predicted by the optimization of fitness (6). For details on the construction of trajectories, see the text. The depth *x* in the graphs is measured in meters.

We further apply the same methodology to find the optimal trajectory of DVM as in the case of an unstructured model using empirical information about the ranking of different DVM trajectories. For the sake of simplicity, we do not list here the estimated coefficients $\lambda_{i}$ based on machine learning. We consider the same two parameterization sets (as in the previous section) for the functions E,S, and G. The resultant graphs of the optimal $\left(x_{1}\left(t\right),x_{2}\left(t\right)\right)$ are shown in Figure 3. The dots in the figures show the empirical data on daily zooplankton migration for adults and young stages [42].

Figure 3a shows the trajectories in the case the functions E,S and *G* are given by linear and quadratic parameterization, i.e., $E=\sigma_{1}\left(x+C\right),S_{x}=\sigma_{2}\left(x+C\right),$ and $G={\left(x+C_{0}\right)}^{2}$. One can prove that the optimal trajectories for both juveniles and adults are given by $x_{1}=A_{1}+B_{1}\cos2\pi t,x_{2}=A_{2}+B_{2}\cos2\pi t,$ where $A_{1},A_{2}$ have the meaning of the average depths through the cycle and $B_{1},B_{2}$ describes the amplitude of vertical migration. Although the mathematical form of the trajectories is the same as in the unstructured population model, for the stage-structured it is impossible to obtain the exact analytical dependence of $A_{1},A_{2},B_{1},B_{2}$, on parameters $M_{i}$. Implementation of machine learning methods gives the following values for the migration amplitude and the mean depths: $A_{2}=-49.2\;m;B_{2}=-46.9\;m;A_{1}=-5.4\;m;B_{1}=-1.568\;m$. Figure 3b shows the optimal trajectories of DVM in the case E,S and *G* are given by hyperbolic functions. For the given values of $\lambda_{i},$ the optimal trajectories were found using the optimal control method similar to those in [19]. For both sets of functional forms of E,S, and G, one can see that early stages of zooplankton do not migrate whereas older stages perform a large-amplitude vertical migration.

Note that compared to the unstructured population model, the system accounting for two or more developmental stages is substantially more complicated, which may result in uncertainty in predictions entirely based on data. The main reason of increasing of uncertainty and sensitivity is the presence of nonlinear terms as well as the increase of the total number of terms in the fitness function. Therefore, it would be helpful to have some additional way of verification of the computational results obtained based on empirical data only. A possible verification can be a comparison of the fitness reconstructed from empirical data and fitness obtained using a trustable analytical model of population dynamics. A possible candidate for a trustable analytical model is a classical two-stage population model based on two coupled differential equations for the densities of juveniles and adults. We should stress that it is a nonlinear model as it includes the effects of intraspecific competition (see Appendix B). For the mentioned model, it is possible to derive the expression of evolutionary fitness analytically. By applying the theoretical model, we assume that it can provide a reasonably good approximation of the underlying ecological process. We also assume that the parameters r,s,p, and *q* in the analytical model describing, respectively, the reproduction, the mortality rate of juveniles, the maturation of juveniles, and the mortality rate of adults can be expressed as the following linear combination of $M_{i}$,

(7) $$p=\theta_{1}M_{1}+\phi_{1}M_{3}+\psi_{1}M_{4},\;\;q=\zeta_{1}M_{2},$$

(8) $$r=\theta_{2}M_{5}+\phi_{2}M_{7}+\psi_{2}M_{8},\;\;s=\zeta_{2}M_{6},$$

where the positive coefficients $\theta_{1},\phi_{1},\psi_{1},\zeta_{1},\theta_{2},\phi_{2},\psi_{2},\zeta_{2}$ have the meaning of weights. Note that mathematically, expressions (7)–(8) can be considered as a linear change of variables. However, their use has also some biological rationale, for example, the reproduction is based on the food consumption, which we subtract the cost of migration and losses due to entering in the unfavorable zones.

We can rewrite evolutionary fitness (6) in terms of new parameters p,q,r,s

(9) $$\begin{array}{c} F\approx h_{1}p+h_{2}q+h_{3}r+h_{4}s+h_{11}p^{2}+h_{22}q^{2}+h_{33}r^{2}+h_{44}s^{2}+\\ +h_{12}pq+h_{13}pr+h_{14}ps+h_{23}qr+h_{24}qs+h_{34}rs. \end{array}$$

Substitution of (7–8) in (9) results in the following expression,

(10) $$\begin{array}{c} F=h_{1}\left(\theta_{1}M_{1}+\phi_{1}M_{3}+\psi_{1}M_{4}\right)+h_{2}\left(\zeta_{1}M_{2}\right)+h_{3}\left(\theta_{2}M_{5}+\phi_{2}M_{7}+\psi_{2}M_{8}\right)+h_{4}\left(\zeta_{2}M_{6}\right)+\\ +h_{11}{\left(\theta_{1}M_{1}+\phi_{1}M_{3}+\psi_{1}M_{4}\right)}^{2}+h_{22}{\left(\zeta_{1}M_{2}\right)}^{2}+h_{33}{\left(\theta_{2}M_{5}+\phi_{2}M_{7}+\psi_{2}M_{8}\right)}^{2}+h_{44}{\left(\zeta_{2}M_{6}\right)}^{2}+\\ +h_{12}\left(\theta_{1}M_{1}+\phi_{1}M_{3}+\psi_{1}M_{4}\right)\left(\zeta_{1}M_{2}\right)+h_{34}\left(\theta_{2}M_{5}+\phi_{2}M_{7}+\psi_{2}M_{8}\right)\left(\zeta_{2}M_{6}\right)+\\ +h_{13}\left(\theta_{2}M_{5}+\phi_{2}M_{7}+\psi_{2}M_{8}\right)\left(\theta_{2}M_{5}+\phi_{2}M_{7}+\psi_{2}M_{8}\right)+h_{14}\left(\theta_{1}M_{1}+\phi_{1}M_{3}+\psi_{1}M_{4}\right)\left(\zeta_{2}M_{6}\right)+\\ +h_{23}\left(\zeta_{1}M_{2}\right)\left(\theta_{2}M_{5}+\phi_{2}M_{7}+\psi_{2}M_{8}\right)+h_{23}\left(\zeta_{1}M_{2}\right)\left(\zeta_{2}M_{6}\right). \end{array}$$

We can now compare expressions (6) and (10) to extract the coefficients corresponding to the same terms $M_{i}$ and $M_{i}M_{j}$. Suppose we know the values of $\lambda_{i},$ then we can find the values of $\theta_{1},\phi_{1},\psi_{1},\zeta_{1},\theta_{2},\phi_{2},\psi_{2}$, and $\zeta_{2}$ up to some multiplies, for example, by fixing a number of coefficients $h_{i}$ (e.g., one can choose four parameters $h_{1},h_{2},h_{3},h_{6})$. The other coefficients $h_{i}$ and $h_{ij}$ can be found from the comparison of (6) and (10). Then, using expressions (7)–(8) and already determined values $M_{i}$, we can find the point (p,q,r,s) in the 4D space. Using the analytical expression for the evolutionary fitness provided in Appendix B, we construct the Taylor expansion in the vicinity of this point. We can compare the coefficients in the expansion of the analytical model and those in (9). By modifying $h_{1},h_{2},h_{3},h_{6}$ and using integrational optimization procedure one can fit the coefficients $h_{i}$ and $h_{ij}$ in the expression of fitness (9) and Taylor expansion of the fitness in the analytical model. The convergence of this procedure is guaranteed in the case the analytical model adequately describes the system. The criterion of a successful verification is that all coefficients $h_{i}$ and $h_{ij}$ should be close for both approaches. An example of the comparison between the analytical and our computational method based on recognition of pair is provided in Table 1. The coefficients in the Taylor expansion of fitness in the analytical form were computed in the vicinity of the point r=0.2344;p=0.1195;q=0.0075;s=0.1086. Note that we rescaled the coefficients, so we set r=1.

From the above table, one can see that there is a good match between the proposed computational method of pair ranking recognition and the analytical model of population dynamics. This provides additional support for the method.

## 4. Discussion

There exist two main approaches to biological fitness identification using data. The first one is based on dynamical models of population dynamics. According to this approach, one first needs to estimate the model parameters and then identify fitness from the underlying dynamical system [10,16,42]. The second approach to fitness identification implies direct evaluation of fitness based on collected empirical data [27]. In this respect, we focus on the second approach, which allows us to reveal evolutionary fitness using empirical data which can be partially guided (but not entirely based on) by underlying theoretical models. The methodology is based on the recently developed framework which defines evolutionary fitness in self-replicating systems as a function, which preserves ranking order of strategies and provides a way to find the evolutionarily stable strategy (i.e., unbeatable by other strategies), as the one which maximizes fitness.

The existence of fitness in the system is related to an important characteristic of the system: its entropy. One can show that in the absence of mutations (or where the mutation rate is low), the evolution of the system should decrease the entropy to its minimal value. This can be seen from the following reasoning. The data set used for learning by artificial network consists of *N* strategies, the presence of each strategy $v_{i}$ is described by $\rho (v_{i},t)$, which for simplicity can be considered as the population density. We can always rescale to verify the condition $\sum_{i=1}^{N}\rho \left(v_{i},t\right)=1$. In other words, we have a probabilistic distribution of strategies. We can define the entropy for such distribution given by $H\left(v_{1},\ldots ,v_{N}\right)=-\sum_{i=1}^{N}\rho \left(v_{i},t\right)\ln\rho \left(v_{i},t\right)$. In the case, we define a partial order for comparison of strategies, it was shown that $\rho (v_{i},t)\to 0$ for $v_{i}\neq v^{*},\rho \left(v^{*},t\right)\to 0$ for t→∞. The entropy tends to its minimal value, i.e., zero. The Shanon entropy characterizes the diversity of the system, thus the diversity of the system tends to its minimal value: a single strategy.

A similar interpretation can be provided for the entire space *V* of strategies, where instead of summation one needs to integrate ρ(v,t) over the entire space *V* (in this case we need to introduce a Borel measure μ, see [22] for detail). One can assume that without losing generality that $\int_{V}\rho \left(v,t\right)\mu \left(dv\right)=1$ (which can always be done by rescaling), i.e., ρ(v,t) describes a probabilistic distribution over *V* with respect to measure μ. In this case, we can define the Shannon entropy of the distribution ρ(v,t) via the following integral $H\left(V\right)=-\int_{V}\rho \left(v,t\right)\ln\rho \left(v,t\right)\mu \left(dv\right)$. If the best element $v^{*}$ exists in the system, the selection will result in H(V)→−∞ for t→−∞, in other words, the entropy tends to its smallest value, which can be interpreted as the decrease of diversity in the system. In the study by A. Gorban, it was shown that for an appropriate choice of measure, the decrease of entropy over time will be a monotonic function [43].

To reconstruct fitness function, we implement computational methods of artificial neural networks to efficiently separate pairs of strategies having the opposite ranking order (i.e., v≺w or v≻w). Machine learning seems to be a natural tool to extend the ranking order of strategies based on the available empirical data to some wider ranges of life history traits. The eventual goal is to predict the evolutionarily optimal strategy over all the feasible ranges of life traits and behavioral patterns. We argue that the proposed approach is rather generic and goes beyond theoretical biology, for example, it is applicable in economics and business to predict the eventual result of the competition of produced goods, services, etc. However, reconstructing evolutionary fitness using machine learning has several important caveats that might make it complicated. First, we require a sample of the time series to be sufficiently long. Second, we assume that the set of evolving strategies is not finite but is a certain continuum (e.g., function space). Third, the main task is not to establish ranking order by itself, but by using it to derive fitness function following this order with the final goal being to predicting the evolutionarily stable strategy. Finally, it is often practically hard to validate the appropriate software.

In this paper, we apply our methodology to explore optimal strategies of regular diel vertical migration (DVM) of herbivorous zooplankton in the ocean and lakes. The methodology overall demonstrated great potential in predicting the evolutionarily stable strategies of zooplankton. In this study, we produced a set of distinct daily vertical trajectories of zooplankton using the existing data on vertical under the assumption that the observed trajectory is the optimal one, i.e., for which fitness is maximal. This was ideal for testing the method since we already know the answer to the problem: which trajectory would be the evolutionarily optimal solution. Note that for the biological systems, which are currently experiencing processes of natural selection, the eventual evolutionary outcome is unclear. As a possible extension the current work on zooplankton migration, it would be interesting to consider a large number of reported empirical cases of DVM and to include more complicated theoretical models of zooplankton population growth to better train neural networks.

Reconstruction of evolutionary fitness from empirical data becomes more difficult in the case where the number of parameters $M_{i}$ is large or/and the expression of fitness contains a large number of nonlinear terms. For example, this problem occurs when considering a population with multiple developmental stages: each stage is characterized by a distinct strategy (e.g., Figure 2 and Figure 3). In this case, additional information about the system would be important to ensure that the weights λ in (4) estimated via machine learning provide a reasonable approximation. Implementation of mathematical models of population dynamics can be of great help as they provide an analytical expression for evolutionary fitness. A good match between the Taylor coefficients of the analytical model and those of the fitness function derived from empirical data gives extra support to the latter. On the other hand, using a fitness function constructed from empirical data by the methodology proposed herein can be helpful to justify the implementation of a particular analytical model of population dynamics. Note that apart from the structured model given in Appendix B, one can consider more complex population models, for example, those including delay [22] or considering continuous aging as given by integro-differential equations [22,42]. Combining these models with available DVM data and revealing fitness might be an interesting extension of the current research.

## Figures and Tables

**Table 1:** Comparison of the coefficients of evolutionary fitness function from the analytical form and the computational method using recognition of ranking of pairs.

Coefficient	Analytical Model	Recognition of Pairs
$h_{3}$	1.00	1.00
$h_{1}$	0.6478	0.6332
$h_{2}$	−1.3472	−1.3692
$h_{4}$	−1.4809	−2.4853
$h_{33}$	−2.1204	−2.0251
$h_{34}$	0.1417	0.1436
$h_{23}$	−0.1417	−0.1589
$h_{13}$	4.1073	3.8467
$h_{44}$	4.2301	4.0135
$h_{24}$	−4.2301	−4.7115
$h_{14}$	−3.9474	−3.3190
$h_{22}$	4.2301	4.8529
$h_{12}$	3.9474	−3.9102
$h_{11}$	−4.7742	−5.4857

**Figure A1:**
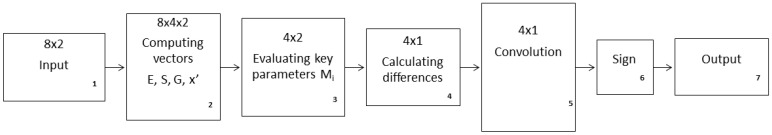
Flowchart of the AI procedure used in the current study to reveal the ranking order of two different strategies of zooplankton DVM.

## Data Availability

Not applicable.

## Appendix A

Here, we provide details on the AI procedure implemented in this study to compare two different strategies of zooplankton DVM. The corresponding flowchart is shown in Figure A1.

The meaning of the numbered boxes in the figure is the following.

1. Input box. Input two vectors *v* and *w* of vertical locations of zooplankton measured every 3 h (determining two different strategies of DVM). The components of these vectors are denoted by $x_{i}\left(v\right)$, $x_{i}\left(w\right)$ with i=1,2,…,8.
2. Evaluation of values of the functions *E*, *S*, *G* as well as the velocity $x^{\prime }$ for both strategies *v* and *w* at the considered discrete points $x_{i}\left(v\right)$, $x_{i}\left(w\right)$ with i=1,2,…,8. We calculate the values of $E(x_{i}\left(v\right))$, $S(x_{i}\left(v\right))$, $G(x_{i}\left(v\right))$, ${\left(x_{i}^{\prime }\left(v\right)\right)}^{2}$, $E(x_{i}\left(w\right))$, $S(x_{i}\left(w\right))$, $G(x_{i}\left(w\right))$, ${\left(x_{i}^{\prime }\left(w\right)\right)}^{2}$.
3. Computation of the key parameters $M_{i}$ via summation of components obtained in step 2 with appropriate signs: $M_{1}=\sum_{1}^{8}E\left(x_{i}\left(v\right)\right)$; $M_{2}=-\sum_{1}^{8}{\left(x_{i}^{\prime }\left(v\right)\right)}^{2}$; $M_{3}=-\sum_{1}^{8}S\left(x_{i}\left(v\right)\right)$; $M_{4}=-\sum_{1}^{8}G\left(x_{i}\left(v\right)\right)$ (for *w* the corresponding expressions will be similar).
4. Calculation of the difference between the key parameters $M_{i}\left(v,w\right)=M_{i}\left(v\right)-M_{i}\left(w\right)$, i=1,2,3,4.
5. Computation of convolution of the differences $M_{i}\left(v,w\right)$ with the corresponding weighting coefficients as $\alpha M_{1}\left(v,w\right)+\gamma M_{2}\left(v,w\right)+\beta M_{3}\left(v,w\right)+\delta M_{4}\left(v,w\right)$.
6. Implementation of a sigmoid function to the convolution found in step 5. Comparison with a fixed threshold value.
7. Output box: interpretation of the obtained result as comparison of strategies by concluding if v≻w or w≻v.

## Appendix B

Evolutionary fitness can be analytically derived for an important class of population dynamics models accounting for age structure [18,22] given by the following equation,

$$z_{t}^{\prime }=L\left(v\right)z-f\left(t,z\right)z,$$

where $z\left(v,t\right)=(z_{1}\left(t,v\right),z_{2}\left(t,v\right),\ldots ,z_{N}\left(t,v\right))$ is a vector describing the population size (density) of each group with the strategy *v* (which is a vector itself), L(v) is nxn matrix describing population growth, mortality and aging, *f* is a term which models self-limitation of the population growth for high densities (we assume that *f* does not depend on *v*), this term describes effects of the intraspecific competition within the population. The number *N* can be understood as the number of developmental stages. Using the above model, the relation between ρ(v) (the indicator of the presence of *v* in the system) and *z* can be understood as $\rho \left(v\right)\equiv \sum z_{i}\left(v\right)$.

It was shown that for the above model the evolutionary fitness is given by $J\left(v\right)=\lambda_{1}\left(v\right),$ where $\lambda_{1}\left(v\right)$ is the eigenvalue of L(v) with the largest real part.

In this paper, we consider a two-stage population model. The model equations are given by

$$\frac{dz_{1}\left(t,v\right)}{dt}=r\left(v\right)z_{1}\left(t,v\right)-p\left(v\right)z_{1}\left(t,v\right)-q\left(v\right)z_{1}\left(t,v\right)-f\left(t,z\right)z_{1}\left(t,v\right)$$

$$\frac{dz_{2}\left(t,v\right)}{dt}=p\left(v\right)z_{1}\left(t,v\right)-s\left(v\right)z_{2}\left(t,v\right)-f\left(t,z\right)z_{2}\left(t,v\right)$$

where $z_{1}\left(t,v\right)$ is the population density of the juvenile (non-reproducible) stages of zooplankton; $z_{2}\left(t,v\right)$ is the population density of adults which can reproduce. In both cases, *v* denotes the corresponding inherited strategy. In the model, the coefficients r,s,p, and *q* describe, respectively, the reproduction, mortality of juveniles, maturation of juveniles, and mortality of adults. They are all functions of the strategy v. Note that *J* does not depend on *f*; this fact can be proved for the generic population model settings with *n* stages and an arbitrary matrix *L* (see in [44] for details).

Previously, it was shown that fitness function for the above two-stage population model is given by [22]

$$J=-s\left(v\right)-p\left(v\right)-q\left(v\right)+\sqrt{4r\left(v\right)p\left(v\right)+{\left(p\left(v\right)+q\left(v\right)-s\left(v\right)\right)}^{2}}.$$

It was also demonstrated that evolutionary fitness in the above model does not depend on the initial condition.
